# Down syndrome predisposes to congenital cardiac malformations

**DOI:** 10.17179/excli2019-1783

**Published:** 2019-09-09

**Authors:** Kamleshun Ramphul, Stephanie G. Mejias, Jyotsnav Joynauth

**Affiliations:** 1 Department of Pediatrics, Shanghai Xin Hua Hospital, Shanghai Jiao Tong University School of Medicine, Shanghai, China; 2 University Iberoamericana Unibe School of Medicine, Santo Domingo, Dominican Republic; 3 Children's Hospital Zhejiang University School of Medicine, Hangzhou, Zhejiang, China

## ⁯

***Dear Editor,***

Congenital heart disease (CHD) is the leading cause of death in Down syndrome (DS) children below the age of 2. The prevalence of CHD in DS has been reported to be between 40-63 % in multiple studies (Benhaourech et al., 2016[1]). A retrospective study was performed to evaluate the risks of congenital cardiac defects in DS patients aged less than 21. 

The 2012 Kids´ Inpatient Database (KID), provided by the Healthcare Cost and Utilization Project (HCUP) and sponsored by the Agency for Healthcare Research and Quality, was used to isolate all cases of Down syndrome patients based on International Classification of Diseases, Ninth Revision, Clinical Modification (ICD-9-CM) codes (HCUP Kids' Inpatient Database, 2012[3]; HCUP Clinical Classifications Software (CCS) for ICD-9-CM, 2012[2]). We evaluated further the sample for various congenital cardiac defects including Ventricular Septal Defect (VSD), Tetralogy of Fallot (TOF), Ostium Primum, Ostium Secundum, Common Truncus, Ebstein anomaly, Coarctation of the aorta, and Patent Ductus Arteriosus (PDA). 

The database consisted of 6,675,222 weighted entries, including 24,371 Down syndrome related admissions (Table 1(Tab. 1)). Among the DS patients, 3,173 (13.0 %) had VSD (p<0.01, OR=32.258, 95 % CI: 31.026-33.539), 512 (2.1 %) had TOF (p<0.01, OR=25.120, 95 % CI: 22.927-27.524), 151 (0.6 %) suffered from Ostium Primum defect (p<0.01, OR=129.960, 95 % CI: 104.646-154.032), and 5,071 (20.8 %) had Ostium Secundum defect (p<0.01, OR=21.051, 95 % CI: 20.394-21.728). We also found that 26 DS (0.1 %) patients were diagnosed with common truncus (p<0.01, OR= 7.099, 95 % CI: 4.802-10.493), while 44 (0.2 %) had Ebstein anomaly (p<0.01, OR= 11.263, 95 % CI: 8.333-15.225). 

The prevalence of PDA and coarctation of the aorta were also higher in DS patients. 4,002 (16.4 %) DS patients had PDA (p<0.01, OR= 20.520, 95 % CI: 19.818-21.246) and 261 (1.1 %) were diagnosed with coarctation of the aorta (p<0.01, OR= 12.417, 95 % CI: 10.961-14.068).

The Down syndrome cell adhesion molecule (DSCAM) is involved in adhesion as well as fusion of endocardial cushions. In Down syndrome patients, there is overexpression of the DSCAM gene that leads to an imbalance in the epithelial-mesenchymal transformation. It can also cause a defect in mesenchymal migration and proliferation that eventually causes several congenital heart defects (Marder et al., 2015[4]). AVSDs have also been linked with extracellular matrix anomalies and it is believed that TOF can be a result of multiple anomalies involving ectomesenchymal tissue migration (Moreno Garcia et al., 2000[5]).

While our results confirm the increased risk of multiple congenital cardiac defects in children with Down syndrome, there are some limitations to the use of HCUP database. It does not allow access to investigate follow-ups of patients and can be influenced by input mistakes. 

## Conflict of interest

The authors have no conflict of interest to declare. 

## Ethical approval

The use of the database followed the requirements set by the HCUP and Agency for Healthcare Research and Quality. No other ethical approval was necessary.

## Informed consent

No informed consent was required for this research.

## Figures and Tables

**Table 1:**
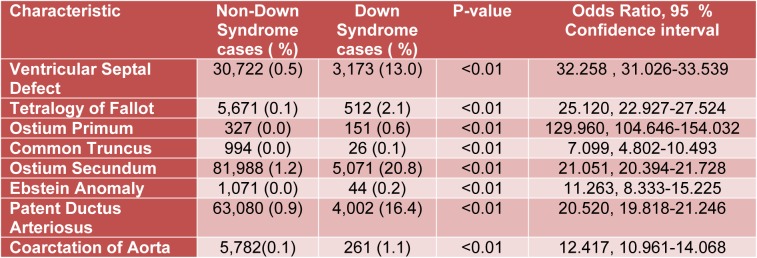
Characteristics of multiple cardiac defects in children with Down syndrome

## References

[1] Benhaourech S, Drighil A, Hammiri AE (2016). Congenital heart disease and Down syndrome: various aspects of a confirmed association. Cardiovasc J Africa.

[2] HCUP Clinical Classifications Software (CCS) for ICD-9-CM (2012). Healthcare Cost and Utilization Project (HCUP). www.hcup-us.ahrq.gov/toolssoftware/ccs/ccs.jsp.

[3] HCUP Kids’ Inpatient Database (KID) (2012). Healthcare Cost and Utilization Project (HCUP). www.hcup-us.ahrq.gov/kidoverview.jsp.

[4] Marder L, Tulloh R, Pascall E (2015). Cardiac problems in Down syndrome. Paediatrics and Child Health.

[5] Moreno Garcia M, Gomez Rodriguez MJ, Barreiro Miranda E (2000). An Esp Pediatr.

